# Future AI Will Most Likely Predict Antibody-Drug Conjugate Response in Oncology: A Review and Expert Opinion

**DOI:** 10.3390/cancers16173089

**Published:** 2024-09-05

**Authors:** Navid Sobhani, Alberto D’Angelo, Matteo Pittacolo, Giuseppina Mondani, Daniele Generali

**Affiliations:** 1 Department of Cancer Biology, The University of Texas MD Anderson Cancer Center, Houston, TX 77030, USA; 2 Department of Medicine, Northern General Hospital, Sheffield S5 7AT, UK; ada43@bath.ac.uk; 3 Department of Surgery, Oncology and Gastroenterology, University of Padova, 35122 Padova, Italy; matteo.pittacolo@aopd.veneto.it; 4 Royal Infirmary Hospital, Foresterhill Health Campus, Aberdeen AB25 2ZN, UK; gmcorno@outlook.com; 5 Department of Medicine, Surgery and Health Sciences, University of Trieste, 34100 Trieste, Italy; dgenerali@units.it

**Keywords:** artificial intelligence, antibody-drug conjugates, prognostic, clinical trials

## Abstract

**Simple Summary:**

This review explores the potential of artificial intelligence (AI) to predict the effectiveness of antibody-drug conjugates (ADCs) in cancer treatment. The problem addressed is the need for more accurate methods to predict how well cancer therapies will work, particularly in personalized medicine. This study’s aim is to discuss how AI can enhance the precision of ADC therapy by analyzing data from clinical trials and molecular biomarkers. This review highlights that AI can significantly reduce the time and cost associated with drug discovery and improve the targeting of cancer cells, reducing side effects and increasing treatment efficacy. We conclude that as more data become available from ongoing clinical trials, AI has the potential to become a standard tool in predicting ADC responses, thereby improving patient outcomes and advancing cancer treatment. This research is valuable as it could lead to more effective and personalized cancer therapies, benefiting society by potentially saving lives and reducing healthcare costs.

**Abstract:**

The medical research field has been tremendously galvanized to improve the prediction of therapy efficacy by the revolution in artificial intelligence (AI). An earnest desire to find better ways to predict the effectiveness of therapy with the use of AI has propelled the evolution of new models in which it can become more applicable in clinical settings such as breast cancer detection. However, in some instances, the U.S. Food and Drug Administration was obliged to back some previously approved inaccurate models for AI-based prognostic models because they eventually produce inaccurate prognoses for specific patients who might be at risk of heart failure. In light of instances in which the medical research community has often evolved some unrealistic expectations regarding the advances in AI and its potential use for medical purposes, implementing standard procedures for AI-based cancer models is critical. Specifically, models would have to meet some general parameters for standardization, transparency of their logistic modules, and avoidance of algorithm biases. In this review, we summarize the current knowledge about AI-based prognostic methods and describe how they may be used in the future for predicting antibody-drug conjugate efficacy in cancer patients. We also summarize the findings of recent late-phase clinical trials using these conjugates for cancer therapy.

## 1. Introduction

Many aspects of society have been influenced by the recent advancements in artificial intelligence (AI). Medicine is one field with the potential for a gradual revolution through the use of AI in the development of drugs and their implementation in clinical trials, stratification of patients for treatment, and prediction of response to cancer therapy. Overall, the purpose of AI in medicine is to reduce humans’ workload while achieving objectives more effectively. It fits in all aspects of medicine, ranging from communication and managerial organization to aiding the more complex issue of selecting therapies for patients.

AI primarily functions through Machine Learning (ML). Deep learning (DL) is a subset of ML that employs artificial neural networks. DL involves more sophisticated and interconnected elements than ML, which resemble electrical impulses in the human brain [1]. When artificial neural networks receive an input, they are trained based on it and use single or multiple linked algorithms to solve problems [2]. The three types of artificial neural networks are multilayer perceptron networks, recurrent neural networks, and convolutional neural networks. They use either supervised or unsupervised training procedures [2,3].

Pharmaceutical companies have used these new AI technologies recently for faster testing of new drugs [4]. Worth noting is that newly discovered drugs have been ranked based on efficacy values (IC_50_ and binding affinity) through molecular simulations and ultimately via in vitro validation experiments [5,6]. This could be used to discover new drugs more efficiently. Therefore, feeding such AI databases could derive more powerful and targeted pharmaceutical products [5,6].

Historically, the process of drug development has been very slow and expensive. The steps from initiation of a drug discovery program to approval by a national drug regulatory agency take 12–15 years [1]. Also, the average cost to bring a drug to the market is USD 2.5 billion [7]. Demonstration of the effectiveness of AI-based methods in shortening these times and reducing these costs in future clinical trials will prove their validity. Recently, a Boston Consulting Group investigation evinced that AI could cut drug discovery costs and time by 25–50% up to the clinical testing stage and that in a 2022 analysis, 20 AI-intensive companies had developed 158 drug candidates compared with 333 candidates developed by other 20 big pharmaceutical companies, which are the world’s largest pharmaceutical companies [4]. This provides a glimpse at how fast this field is evolving and the way it could be used to predict therapy efficacy holds immense implications.

In contrast with conventional chemotherapy, which can damage healthy cells, antibody-drug conjugates (ADCs) deliver chemotherapeutic agents to cancer cells more specifically [8]. ADCs rely on a monoclonal antibody’s recognition of a specific receptor target expressed on the surface of cancer cells. And after its binding ADC is internalized by the cell, the ADC then releases the cytotoxic drug via a linker attached to the antibody inside the cancer cell, permitting the specific release of the drug to the cancer cells. Fully human monoclonal antibodies are highly targeted, have long circulating half-lives, and have low immunogenicity. The role of the linker in this process is paramount because they should firmly keep the payload bound to the antibody. These drug conjugates should be constructed to be stable enough to prevent cleavage of the linker before they become internalized in cancer cells [8,9]. If the payload is accidentally released before reaching its target, it could cause toxicity. Among the benefits of this type of therapy related to the specificity of antibody-receptor recognition is a reduction in toxicity because much fewer normal cells are targeted than in conventional chemotherapy. Therefore, dose escalation could be more easily performed using ADCs, enhancing the efficacy of treatment [10]. Currently, 13 ADCs are approved by the U.S. Food and Drug Administration (FDA), and 100 are going through clinical trials [10].

In this review, we summarize the current knowledge about AI-based prognostic methods and describe how they may be used in the future for predicting antibody-drug conjugate efficacy in cancer patients. We also summarize findings of recent late-phase clinical trials using these conjugates for cancer therapy.

## 2. Prediction of Cancer Responsiveness and Resistance to ADCs

Various AI methods have been developed to develop new cancer drugs, cancer prognoses, and responses to cancer therapies. These technologies are discussed below to show how they can potentially be employed in the construction of new AI algorithms for the use of ADCs, specifically, in identifying potential challenges in the field of oncology and cancer therapy selection and determining how they could be solved based on the knowledge generated in other related fields where AI has produced promising results.

Because drug discovery is beyond the scope of this review, we mention only a few to explain how they are being employed in medical research around the world. The mainstream AI methods employed for drug discovery use a wide variety of data resources, such as ChEMBL and DrugBank. After the drugs’ potential efficacy is ranked, their toxicity, bioactivity, and physicochemical properties are ranked [11]. Interestingly to ADC drug discovery, the Response Algorithm for Drug Positioning and Rescue (Lantern Pharma) is an AI platform capable of rapidly developing novel ADCs, including cryptomycin-derived ADCs.; AtomNet is another effective technology predicting the binding activity of novel chemicals to their intended therapeutical targets [12]. Various AI-based tools are capable of identifying the physicochemical properties of drugs. Each pharmaceutical company may have a patent-protected AI drug discovery method, which complicates the comparison of the methods. These technologies integrate data from preclinical and clinical tests, such as data in CellMinerCDB with The Cancer Genome Atlas, the Catalogue of Somatic Mutations in Cancer, the Gene Expression Omnibus, and identify published articles to generate new insights into the drug structures and targeting of proteins of interest [13,14,15,16,17]. A more comprehensive review of AI drug discovery methods was performed by Paul et al. [1].

Conceivably, these algorithms and databases could be adapted to test ADC responsiveness during clinical trials. In this review we would like to share our expert opinion on how the technologies of AI could be employed in the near future to generate a self-learning algorithm, based on the information provided from the current clinical trials, to best predict the outcomes. Once hopefully, properly tested, validated and consolidated, having such an incredible technology at hand could be of paramount importance to guide clinicians best decide on which course of therapy would be most effective without incurring errors. Of note is that the potential of an AI system depends on the quality of the data used to feed the ML process. Table 1 summarizes the current databases that could be used to create AI models for cancer therapy response prediction and drug design. With the accrual of information from clinical studies on molecular biomarkers in tumor tissue, circulating tumor DNA, or circulating cell-free DNA, more data are generated that could help to predict the responsiveness of cancer to therapy; having AI systems to help process such data more efficiently would be beneficial [18,19,20,21]. This could result in the provision of real-time information to physicians regarding the potential responsiveness of cancer to ADCs and what courses of action could be planned in case a drug is statistically likely to fail in a specific case.

Currently, AI-aided methods of cancer prognosis have demonstrated notable advances when compared with image-based prognosis. For example, the combination of radiomics and AI has successfully extracted and processed multidimensional data from cancer images, such as magnetic resonance imaging, computed tomography, ultrasound (US), digital subtraction angiography, and X-ray images [22]. For hepatocellular carcinoma (HCC) patients, AI coupled with radiomics has shown the potential to improve tumor characterization and offer a better prognosis than conventional radiological methods. This coupling yields insights into the complex relationship between radiomic variables and clinical outcomes [23]. The process of automatic segmentation in programming ML, which delineates the volume of interest, could help predict treatment response [24,25]. Also, DL can bypass the conventional steps of ML radiomic analysis. The output is calculated via DL through filtering and calculations of unprocessed images of HCC lesions serving as inputs. The outputs can include prediction of response or non-response to treatment. Furthermore, convolutional neural networks are capable of learning, thereby increasing the accuracy of their overall prediction of ML [26]. Notably, DL can incorporate time as a variable during the evaluation of lesion enhancement patterns in images [27,28]. DL requires more computational power than ML and is more dependent on training with large data sets and a variety of data. DL has greater potential than ML to predict the response of cancers to therapy. In the future, this could be used for ADC-based therapy response prediction as well.

Zhang et al. used a DL system to make an automatic tumor segmentation model capable of integrating clinical variables and preprocedural digital subtraction angiography videos to predict the response of ADCs to transarterial chemoembolization [27]. The authors observed a marked difference in the 3-year progression-free survival rate between responders and non-responders with their fully automated framework (DSA-Net). Their DSA-Net entails a U-net model employed to automate tumor segmentation (Model 1) and a ResNet model that is used to predict response to therapy to the first TACE (Model 2). Both models were tested in 360 patients. For validation, 124 internal patients and 121 external patients’ data were used. Also, Peng et al. [29] developed a PyRadiomics method to predict the response of TACE treatment based on a conventional ML model that was capable of predicting the initial response of cancer to transarterial chemoembolization by exploiting pretreatment computed tomography images. They showed that patients predicted to be treatment responders had longer progression-free and overall survival than predicted non-responders. Additionally, Peng and colleagues applied this model to 46 HCC patients with data in The Cancer Genome Atlas to analyze the differential gene expression across their cohort and the TCGA-HCC cohort to explore the potential mechanisms of action of transarterial chemoembolization. They further used ML to incorporate TCGA genetic data into their data, again showing how versatile this ML method can be in processing large data sets.

Researchers have also examined post-ablation prognosis for cancer therapy using AI. For example, Ma et al. compared the performance of a DL model trained using contrast-enhanced US (CEUS) with that of a conventional ML model trained using static US to predict HCC recurrence after ablation. As expected, the DL model outperformed the ML model, possibly because CEUS, besides providing morphological images, can provide real-time dynamic blood perfusion information that correlates well with the success of ablation [28].

In addition, Liu et al. used clinical data as well as features extracted from CEUS images to predict the 2-year progression-free survival rate in early-stage HCC patients who underwent radiofrequency ablation and surgical resection as well as to determine the optimal treatment for these patients. They found that 17.3% and 27.3% of the patients receiving radiofrequency ablation and surgical resection, respectively, would have had better outcomes if they had received the other treatment instead. A multicenter study with more patients is needed to determine the statistical power of this study. However, this study still demonstrates the potential of AI methods in selecting optimal ADC-based treatments for cancer patients [10].

Despite the encouraging findings, these image-based AI methods require further testing and standardization before they can be effectively integrated into clinical practice. They are operator-dependent and involve different machines, variables, and contrast doses as well as timing [30].

These and similar AI models used for cancer prognostication must be improved to ensure safe and effective patient care. They also must be submitted for and receive FDA approval before implementation in clinical settings. Recently, the FDA proposed a pathway that could lead to the use of ML software applications as medical devices [31]. The AI model should include the following: (1) good ML practice, which means it should be evidence-based for reproducibility purposes, have standardized steps (e.g., the extraction algorithms), use different time points to permit generalizability, and have the consistency of AI analysis and increase the operability across clinical institutions around the world; (2) avoidance of algorithm biases, which should be ensured by validating the testing process with external data to confirm the generalizability of the model; and (3) transparency of the AI models’ logic, which could be achieved by clearly explaining the mechanisms of the AI decision-making process and familiarizing oncologists with these new models [22,32,33,34,35,36].

Standardization of the protocols can be achieved by specifically following commonly approved steps and protocols. One such step is having open databases where previous ADC data could be stored and made available for training purposes.

For decades, prediction tools have been used to support clinical decisions regarding therapy selection, including the ABCD score, the Framingham Risk Score, the Model for End-Stage Liver Disease, and the Nottingham Prognostic Index. In recent years, hundreds of more prediction model studies have appeared [37,38,39,40,41,42]. To prevent the scientific community from becoming mesmerized by the AI revolution and to enable ML prediction models to be appropriately developed, tested, and, if needed, tailored to different contexts before they can be employed in daily medical practice, steps have been taken. In response, new methods have been deemed necessary to resolve the issue of incomplete reporting of models in prediction model studies [43,44]. Specifically, the Transparent Reporting of a multivariable prediction model for Individual Prognosis or Diagnosis (TRIPOD) method was designed to guide the key items to report in new studies or update clinical prediction models [45,46,47]. In AI-based discovery of medical diagnosis, one must also consider that some FDA-approved, clinician-free, AI-based imaging diagnostic tools used for the identification of wrist fractures and strokes in adults have given false diagnoses [48]. This shows the importance of having methods to facilitate the organic, healthy development of new AI-based prognostic methods. It also shows how today’s AI is not unfailing.

Previously, the TRIPOD method was based on the use of regression models. However, a new TRIPOD initiative specific to ML has been developed. This initiative aims to use ML prediction algorithms to establish long-term standardized methodologies for the prediction of prognostic and diagnostic prediction models. New guidelines for the efficient use of prognostic models should be made available with the TRIPOD-Artificial Intelligence (TRIPOD-AI) tool and the Prediction model Risk of Bias Assessment Tool-Artificial Intelligence (PROBAST-AI) [49]. These guidelines are valuable for many AI-based prognostic models, including future methods to predict ADC efficacy. TRIPOD-AI and PROBAST-AI are being developed following guidance from the EQUATOR Network, which consists of five stages: (1) two systematic reviews to examine the quality of the published ML prediction model studies, (2) consultation with key stakeholders using the Delphi method to identify items that should be included in the method, (3) virtual consensus meetings to consolidate and prioritize the key items to be included, (4) development of a TRIPOD-AI checklist and the PROBAST-AI tool, and (5) dissemination of information about the new written algorithms the TRIPOD-AI and PROBAST-AI in journals, conferences, and social media [49].

Another field in which AI has recently shown great promise is cancer immunotherapy. Immunotherapy consists of controlling and eliminating tumors in the human body by eliciting the body’s immune system against cancer, leading to an antitumor immune response. The two main cancer immunotherapy types are immune checkpoint blockade and adoptive cell therapy [50]. AI technology can be used for neoantigen recognition, antibody design, and immunotherapy response prediction [51]. Also, AI can be used to predict new tumor antigens in patients’ cancer rapidly and accurately, reducing experimental screening and validation costs. AI-enhanced antibodies that have the potential for further success than conventional therapies in cancer treatment can be developed. Finally, AI can be used to identify patients whose disease may respond to immunotherapy using multimodal, multiscale biomarkers and immune microenvironments feeding the algorithms for prediction [51].

## 3. Anticancer ADCs That Have Entered Clinical Trials

Years of research and refinement, significant technological advancements, and a deeper understanding by the scientific community of ADC mechanisms have culminated in the FDA’s approval of 11 ADCs, each offering tangible benefits to cancer patients. Among them, fam-trastuzumab deruxtecan-nxki (Enhertu) stands out, as it is poised to capture a substantial market share within the ADC landscape. Its versatility in treating various breast cancer subsets (HER2+, HR+/HER2−, and triple-negative) and extended treatment duration underscore its potential positive impact on breast cancer therapy.

Despite the inherent risks associated with drug development, the trajectory of novel anticancer therapies suggests an imminent surge in ADC approvals. Whether through the introduction of novel ADCs or chemical modification of previous drugs, the outlook for ADC-based cancer therapy is promising. Since the inception of the first ADC clinical trial in 1997, the field has witnessed remarkable proliferation, with 266 additional ADCs undergoing evaluation in more than 1200 clinical trials. This surge indicates a paradigm shift toward targeted cancer therapy.

**Figure 1:**
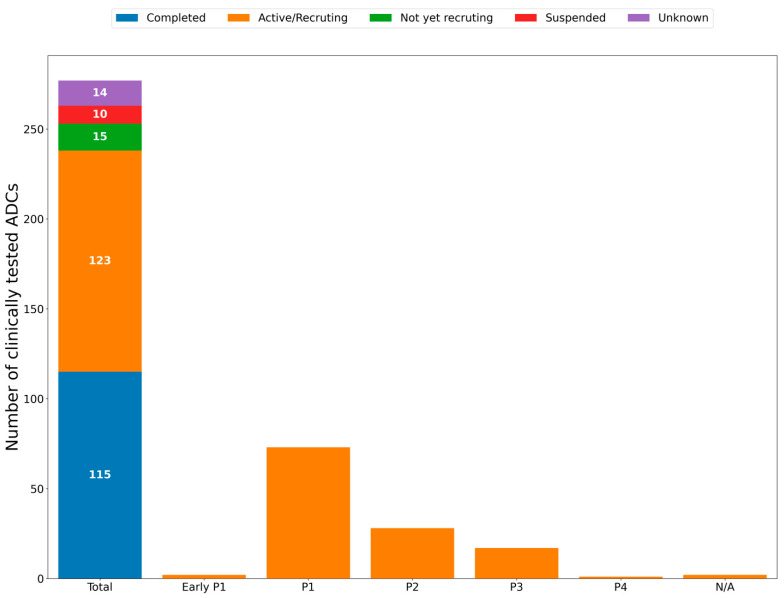
Clinically tested ADCs. This bar graph shows the 277 ADCs that have undergone clinical trials along with their trial status (completed, active/recruiting, not yet recruiting, suspended, and unknown). Additionally, to the right of the main Total bar, the active/recruiting ADCs are broken down into additional columns to highlight their highest developmental stage (phases 1–4 [P1–P4]).

**Figure 2:**
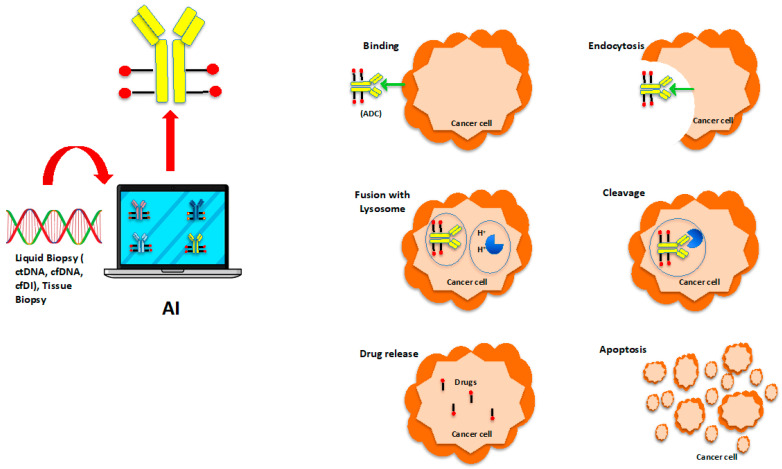
Artificial intelligence assisted antibody-drug conjugate selection for the treatment of cancer.

Presently, 275 clinical ADC trials are active (Table 2), in which investigators are testing different ADCs for accurate delivery of cytotoxic agents (Figure 1), which in the future could be achieved with the help of AI (Figure 2). Notably, discontinued ADCs also underwent rigorous clinical testing, reflecting the commitment to scientific rigor and patient safety regarding treatment with these agents.

Although cancer has served as the proving ground for ADC-based therapies, their applicability across diverse medical domains is increasingly being recognized. With growing interest from major pharmaceutical companies, the ADC market is poised for sustained expansion, fueling optimism for the emergence of blockbuster ADCs in the near future. The use of AI to predict their response poses a hopeful avenue across the different and difficult medical domains.

## 4. Discussion

Over the past decade, advances in AI have pushed the boundaries of the medical field [1,22]. Despite the successful development and use of AI-based diagnostic tools for prediction of cancer treatment response, response to certain targeted therapies remains unpredictable. However, in the field of ADCs, in which cancer patients are stratified for treatment based on the expression of a receptor on the cancer cell membrane that can be specifically bound by an antibody carrying the cytotoxic payload, more accurate prognostic methods that can predict whether patients’ disease would respond to ADCs are needed. ML has shown great potential in many fields, such as radiology or mammography, for early breast cancer detection. It can be used to predict the chemistry of novel compounds against cancer. For such reasons, AI models could play an important role in this prediction of ADC response in the future. Data from ADC clinical trials are always becoming more available biomarkers retrieved from liquid biopsy from circulating tumor DNA, cell-free DNA, tissue samples, or even the tumor microenvironment. Such data could be of paramount importance to feed new AI models to predict ADC therapy [18,19,20,21]. This review has limitations as the method of AI-based ADCs therapy response is still in its conceptual early stage. However, in this review, we summarize the current knowledge in this complex field, ranging from AI models for chemical structure prediction to ongoing clinical trials testing ADCs, without implementing AI now. We hope that the knowledge we have summarized here could serve as a useful tool for generating new AI models in the future based on our hypothesis. Based on our knowledge, using AI models could be paramount for the prediction of ADC efficacy in the near future. As technology advances, it would be impossible to think that such achievements would leave out the field of medicine, in particular oncology, where there is a lot of hope filling lives [52].

The implementation of new AI models, similar to the ones currently available for other prognostic models, would need the close collaboration of software engineers, data scientists, and decision-making medical doctors and scientists. The first step would be for software engineers to go through the different AI systems and modify them into systems for ADC therapy prediction. Once the data science has been sorted, the contribution of the medical doctors would be to provide information on response to therapy and blood-based biomarkers, or even breath-based biomarkers, from ongoing and completed clinical trials involving ADC in diseases. Secondly, the prediction system should be tested in a small subset of cancer patients. The data generated should be used to train the machine learning for its refinement. Thirdly, the model would be tested in a larger cohort of patients. After all these steps have been completed, the method could be commercialized. The coming of new brave ideas will require a shift in the way we are thinking medicine.

## 5. Conclusions

While AI has been implemented in different fields, ranging from the prediction of chemical structure and diagnostic in radiology to other aspects of society, there is a lack of tests for the prognostic of targeted therapies, such as ADCs in oncology at the moment. As these technologies become more popular, more data from clinical trials such as from the summarized ADC clinical trials, become more widely accessible, we envision that such methods will become part of the standard of care.

## Figures and Tables

**Table 1:** Current database resources that could be used for building AI models for therapy prediction.

Name	Main Features	Web Link
CGHub	Cancer genomics data repository	https://docs.gdc.cancer.gov/Encyclopedia/pages/Cancer_Genomics_Hub/ (accessed on 2 September 2024)
TCGA	Comprehensive database of cancer patients’ genomic, epigenomic, transcriptomic, and proteomic data.	https://www.cancer.gov/ccg/research/genome-sequencing/tcga (accessed on 2 September 2024)
CCLE	Comprehensive genetic database of cancer cell lines	https://sites.broadinstitute.org/ccle (accessed on 2 September 2024)
EGA	European genetic, phenotypic, and clinical data repository	https://ega-archive.org/ (accessed on 2 September 2024)
DepMap	High data quality visualization tool	https://depmap.org/portal/ (accessed on 2 September 2024)
SomamiR	Cancer somatic mutation and miRNA correlation	https://compbio.uthsc.edu/SomamiR/ (accessed on 2 September 2024)
COSMIC	Comprehensive somatic mutation database	https://cancer.sanger.ac.uk/cosmic (accessed on 2 September 2024)
MethyCancer	DNA methylations, cancer-related genes, and mutations in correlation with additional cancer information	http://methycancer.psych.ac.cn/ (accessed on 2 September 2024)
CTRP	connecting genetic, cellular features, lineage to cancer cell-lines sensitivity to small molecules	https://portals.broadinstitute.org/ctrp/ (accessed on 2 September 2024)
gCSI	Large number of transcriptomics data	https://pharmacodb.pmgenomics.ca/datasets/4 (accessed on 2 September 2024)
GDSC	Drug response, including genomics markers of drug sensitivity	https://www.cancerrxgene.org/ (accessed on 2 September 2024)
NCI60	Large number of drug and genomics data	https://discover.nci.nih.gov/cellminer/loadDownload.do (accessed on 2 September 2024) https://dtp.cancer.gov/databases_tools/bulk_data.htm (accessed on 2 September 2024)
canSAR	Comprehensive drug discovery database	https://cansarblack.icr.ac.uk/ (accessed on 2 September 2024)
cBioPortal	Large database of cancer genomics data	https://www.cbioportal.org/datasets (accessed on 2 September 2024)
UCSC	Synthetical genomics information	https://genome.ucsc.edu/ (accessed on 2 September 2024)
dbNSFP	Non-synonymous single-nucleotide variants	https://sites.google.com/site/jpopgen/dbNSFP (accessed on 2 September 2024)
NONCODE	Non-coding RNAs database	http://www.noncode.org/ (accessed on 2 September 2024)
TCIA	Comprehensive immunogenomic data from the NGS of 20 solid tumors from TCGA	https://www.tcia.at/home (accessed on 2 September 2024)
ARCHS4	Comprehensive RNA-Sequenced data from human and mouse	https://maayanlab.cloud/archs4/ (accessed on 2 September 2024)

**Table 2:** List of active Phase III clinical trials investigating an antibody-conjugated drug in solid and blood malignancies.

NCT Number	Study Title	Study URL	Study Status	Conditions	ADC	Sponsor
NCT06340568	A Clinical Study of the Anti-cancer Effects of an Investigational Therapy or Chemotherapy in Patients With Recurring Uterine Cancer	https://clinicaltrials.gov/study/NCT06340568 (accessed on 2 September 2024)	Not yet recruiting	Endometrial Cancer	DRUG: BNT323/DB-1303|DRUG: Doxorubicin|DRUG: Paclitaxel	BioNTech SE
NCT05609968	Study of Pembrolizumab (MK-3475) Monotherapy Versus Sacituzumab Govitecan in Combination With Pembrolizumab for Participants With Metastatic Non-small Cell Lung Cancer (NSCLC) With Programmed Cell Death Ligand 1 (PD-L1) Tumor Proportion Score (TPS) ‚ ≥50% (MK-3475-D46)	https://clinicaltrials.gov/study/NCT05609968 (accessed on 2 September 2024)	Recruiting	Carcinoma|Non-Small Cell Lung Cancer	BIOLOGICAL: Sacituzumab govitecan|BIOLOGICAL: Pembrolizumab	Merck Sharp & Dohme LLC
NCT03529110	DS-8201a Versus T-DM1 for Human Epidermal Growth Factor Receptor 2 (HER2)-Positive, Unresectable and/or Metastatic Breast Cancer Previously Treated With Trastuzumab and Taxane [DESTINY-Breast03]	https://clinicaltrials.gov/study/NCT03529110 (accessed on 2 September 2024)	Active—Not yet recruiting	Breast Cancer	DRUG: Trastuzumab deruxtecan (T-DXd)|DRUG: Ado-trastuzumab emtansine (T-DM1)	Daiichi Sankyo
NCT06203210	A Study of Ifinatamab Deruxtecan Versus Treatment of Physician’s Choice in Subjects With Relapsed Small Cell Lung Cancer	https://clinicaltrials.gov/study/NCT06203210 (accessed on 2 September 2024)	Not yet recruiting	Small Cell Lung Cancer	DRUG: Ifinatamab deruxtecan|DRUG: Topotecan|DRUG: Amrubicin|DRUG: Lurbinectedin	Daiichi Sankyo
NCT02631876	A Study of Mirvetuximab Soravtansine vs. Investigator’s Choice of Chemotherapy in Women With Folate Receptor (FR) Alpha Positive Advanced Epithelial Ovarian Cancer (EOC), Primary Peritoneal or Fallopian Tube Cancer	https://clinicaltrials.gov/study/NCT02631876 (accessed on 2 September 2024)	Completed	Epithelial Ovarian Cancer|Primary Peritoneal Carcinoma|Fallopian Tube Cancer|Ovarian Cancer	DRUG: Mirvetuximab soravtansine|DRUG: Paclitaxel|DRUG: Pegylated liposomal doxorubicin|DRUG: Topotecan	ImmunoGen, Inc.
NCT03734029	Trastuzumab Deruxtecan (DS-8201a) Versus Investigator’s Choice for HER2-low Breast Cancer That Has Spread or Cannot be Surgically Removed [DESTINY-Breast04]	https://clinicaltrials.gov/study/NCT03734029 (accessed on 2 September 2024)	Active—Not yet recruiting	Breast Cancer	DRUG: Trastuzumab deruxtecan (DS-8201a)|DRUG: Capecitabine|DRUG: Eribulin|DRUG: Gemcitabine|DRUG: Paclitaxel|DRUG: Nab-paclitaxel	Daiichi Sankyo
NCT04494425	Study of Trastuzumab Deruxtecan (T-DXd) vs. Investigator’s Choice Chemotherapy in HER2-low, Hormone Receptor Positive, Metastatic Breast Cancer	https://clinicaltrials.gov/study/NCT04494425 (accessed on 2 September 2024)	Active—Not yet recruiting	Advanced or Metastatic Breast Cancer	DRUG: Trastuzumab deruxtecan|DRUG: Capecitabine|DRUG: Paclitaxel|DRUG: Nab-Paclitaxel	AstraZeneca
NCT04595565	Sacituzumab Govitecan in Primary HER2-negative Breast Cancer	https://clinicaltrials.gov/study/NCT04595565 (accessed on 2 September 2024)	Recruiting	HER2-negative Breast Cancer|Triple Negative Breast Cancer	DRUG: Capecitabine|DRUG: Carboplatin|DRUG: Cisplatin|DRUG: Sacituzumab govitecan	German Breast Group
NCT05687266	Phase III, Open-label, First-line Study of Dato-DXd in Combination With Durvalumab and Carboplatin for Advanced NSCLC Without Actionable Genomic Alterations	https://clinicaltrials.gov/study/NCT05687266 (accessed on 2 September 2024)	Recruting	NSCLC	DRUG: Datopotamab deruxtecan|DRUG: Durvalumab|DRUG: Carboplatin|DRUG: Pembrolizumab|DRUG: Cisplatin|DRUG: Pemetrexed|DRUG: Paclitaxel	AstraZeneca
NCT05104866	A Phase-3, Open-Label, Randomized Study of Dato-DXd Versus Investigator’s Choice of Chemotherapy (ICC) in Participants With Inoperable or Metastatic HR-Positive, HER2-Negative Breast Cancer Who Have Been Treated With One or Two Prior Lines of Systemic Chemotherapy (TROPION-Breast01)	https://clinicaltrials.gov/study/NCT05104866 (accessed on 2 September 2024)	Active—Not yet recruiting	Breast Cancer	DRUG: Dato-DXd|DRUG: Capecitabine|DRUG: Gemcitabine|DRUG: Eribulin|DRUG: Vinorelbine	AstraZeneca
NCT06161025	A Study of Raludotatug Deruxtecan (R-DXd) in Subjects With Platinum-resistant, High-grade Ovarian, Primary Peritoneal, or Fallopian Tube Cancer	https://clinicaltrials.gov/study/NCT06161025 (accessed on 2 September 2024)	Recruiting	Solid Cancer	DRUG: R-DXd|DRUG: Gemcitabine|DRUG: Paclitaxel|DRUG: Topotecan|DRUG: PLD	Daiichi Sankyo
NCT04639986	Asian Study of Sacituzumab Govitecan (IMMU-132) in HR+/HER2− Metastatic Breast Cancer (MBC)	https://clinicaltrials.gov/study/NCT04639986 (accessed on 2 September 2024)	Active—Not yet recruiting	Metastatic Breast Cancer	DRUG: Sacituzumab govitecan-hziy|DRUG: Eribulin mesylate injection|DRUG: Capecitabine oral product|DRUG: Gemcitabine injection|DRUG: Vinorelbine injection	Gilead Sciences
NCT04296890	A Study of Mirvetuximab Soravtansine in Platinum-Resistant, Advanced High-Grade Epithelial Ovarian, Primary Peritoneal, or Fallopian Tube Cancers With High Folate Receptor-Alpha Expression	https://clinicaltrials.gov/study/NCT04296890 (accessed on 2 September 2024)	Completed	Epithelial Ovarian Cancer|Peritoneal Cancer|Fallopian Tube Cancer	DRUG: Mirvetuximab soravtansine	ImmunoGen, Inc.
NCT01100502	A Phase 3 Study of Brentuximab Vedotin (SGN-35) in Patients at High Risk of Residual Hodgkin Lymphoma Following Stem Cell Transplant (The AETHERA Trial)	https://clinicaltrials.gov/study/NCT01100502 (accessed on 2 September 2024)	Completed	Disease, Hodgkin	DRUG: Brentuximab vedotin|DRUG: Placebo	Seagen Inc.
NCT06103864	A Phase III Study of Dato-DXd With or Without Durvalumab Compared With Investigator’s Choice of Chemotherapy in Combination With Pembrolizumab in Patients With PD-L1 Positive Locally Recurrent Inoperable or Metastatic Triple-negative Breast Cancer	https://clinicaltrials.gov/study/NCT06103864 (accessed on 2 September 2024)	Recruiting	Breast Cancer	DRUG: Dato-DXd|DRUG: Durvalumab|DRUG: Paclitaxel|DRUG: Nab-paclitaxel|DRUG: Gemcitabine|DRUG: Carboplatin|DRUG: Pembrolizumab	AstraZeneca
NCT01712490	A Frontline Therapy Trial in Participants With Advanced Classical Hodgkin Lymphoma	https://clinicaltrials.gov/study/NCT01712490 (accessed on 2 September 2024)	Active—Not yet recruiting	Hodgkin Lymphoma	DRUG: Brentuximab vedotin|DRUG: Doxorubicin|DRUG: Bleomycin|DRUG: Vinblastine|DRUG: Dacarbazine	Takeda
NCT05622890	A Single-arm Clinical Trial of IMGN853 in Chinese Adult Patients With Platinum-resistant, Epithelial Ovarian Cancer	https://clinicaltrials.gov/study/NCT05622890 (accessed on 2 September 2024)	Recruiting	Epithelial Ovarian Cancer|Peritoneal Cancer|Fallopian Tube Cancer	DRUG: Mirvetuximab soravtansine	Hangzhou Zhongmei Huadong Pharmaceutical Co., Ltd.
NCT06112379	A Phase III Randomised Study to Evaluate Dato-DXd and Durvalumab for Neoadjuvant/Adjuvant Treatment of Triple-Negative or Hormone Receptor-low/HER2-negative Breast Cancer	https://clinicaltrials.gov/study/NCT06112379 (accessed on 2 September 2024)	Recruiting	Breast Cancer	DRUG: Dato-DXd|DRUG: Durvalumab|DRUG: Pembrolizumab|DRUG: Doxorubicin|DRUG: Epirubicin|DRUG: Cyclophosphamide|DRUG: Paclitaxel|DRUG: Carboplatin|DRUG: Capecitabine|DRUG: Olaparib	AstraZeneca
NCT04209855	A Study of Mirvetuximab Soravtansine vs. Investigator’s Choice of Chemotherapy in Platinum-Resistant, Advanced High-Grade Epithelial Ovarian, Primary Peritoneal, or Fallopian Tube Cancers With High Folate Receptor-Alpha Expression	https://clinicaltrials.gov/study/NCT04209855 (accessed on 2 September 2024)	Active—Not yet recruiting	Epithelial Ovarian Cancer|Peritoneal Cancer|Fallopian Tube Cancer	DRUG: Mirvetuximab soravtansine|DRUG: Paclitaxel|DRUG: Topotecan|DRUG: Pegylated liposomal doxorubicin	ImmunoGen, Inc.
NCT05751512	A Study to Evaluate MRG003 vs. Cetuximab/Methotrexate in in the Treatment of Patients With RM-SCCHN	https://clinicaltrials.gov/study/NCT05751512 (accessed on 2 September 2024)	Not yet recruiting	Squamous Cell Carcinoma of the Head and Neck	DRUG: MRG003|DRUG: Cetuximab injection|DRUG: Methotrexate injection	Shanghai Miracogen Inc.
NCT05374512	A Study of Dato-DXd Versus Investigator’s Choice Chemotherapy in Patients With Locally Recurrent Inoperable or Metastatic Triple-negative Breast Cancer, Who Are Not Candidates for PD-1/PD-L1 Inhibitor Therapy (TROPION-Breast02)	https://clinicaltrials.gov/study/NCT05374512 (accessed on 2 September 2024)	Recruiting	Breast Cancer	DRUG: Dato-DXd|DRUG: Paclitaxel|DRUG: Nab-paclitaxel|DRUG: Carboplatin|DRUG: Capecitabine|DRUG: Eribulin mesylate	AstraZeneca
NCT05629585	A Study of Dato-DXd With or Without Durvalumab Versus Investigator’s Choice of Therapy in Patients With Stage I-III Triple-negative Breast Cancer Without Pathological Complete Response Following Neoadjuvant Therapy (TROPION-Breast03)	https://clinicaltrials.gov/study/NCT05629585 (accessed on 2 September 2024)	Recruiting	Breast Cancer	DRUG: Dato-DXd|DRUG: Durvalumab|DRUG: Capecitabine|DRUG: Pembrolizumab	AstraZeneca
NCT03523585	DS-8201a in Pre-treated HER2 Breast Cancer That Cannot be Surgically Removed or Has Spread [DESTINY-Breast02]	https://clinicaltrials.gov/study/NCT03523585 (accessed on 2 September 2024)	Active—Not yet recruiting	Breast Cancer	DRUG: Trastuzumab deruxtecan|DRUG: Capecitabine|DRUG: Lapatinib|DRUG: Trastuzumab	Daiichi Sankyo
NCT01777152	ECHELON-2: A Comparison of Brentuximab Vedotin and CHP With Standard-of-care CHOP in the Treatment of Patients With CD30-positive Mature T-cell Lymphomas	https://clinicaltrials.gov/study/NCT01777152 (accessed on 2 September 2024)	Completed	Anaplastic Large-Cell Lymphoma|Non-Hodgkin Lymphoma|T-Cell Lymphoma	DRUG: Brentuximab vedotin|DRUG: Doxorubicin|DRUG: Prednisone|DRUG: Vincristine|DRUG: Cyclophosphamide	Seagen Inc.
NCT06074588	MK-2870 Versus Chemotherapy in Previously Treated Advanced or Metastatic Nonsquamous Non-small Cell Lung Cancer (NSCLC) With EGFR Mutations or Other Genomic Alterations (MK-2870-004)	https://clinicaltrials.gov/study/NCT06074588 (accessed on 2 September 2024)	Recruiting	Non-small Cell Lung Cancer (NSCLC)	BIOLOGICAL: MK-2870|DRUG: Docetaxel|DRUG: Pemetrexed	Merck Sharp & Dohme LLC
NCT03474107	A Study to Evaluate Enfortumab Vedotin Versus (vs) Chemotherapy in Subjects With Previously Treated Locally Advanced or Metastatic Urothelial Cancer (EV-301)	https://clinicaltrials.gov/study/NCT03474107 (accessed on 2 September 2024)	Active—Not yet recruiting	Ureteral Cancer|Urothelial Cancer|Bladder Cancer	DRUG: Enfortumab Vedotin|DRUG: Docetaxel|DRUG: Vinflunine|DRUG: Paclitaxel	Astellas Pharma Global Development, Inc.
NCT05754853	A Study of MRG002 Versus Investigator’s Choice of Chemotherapy in the Treatment of Patients With HER2-positive Unresectable Advanced or Metastatic Urothelial Cancer	https://clinicaltrials.gov/study/NCT05754853 (accessed on 2 September 2024)	Recruiting	Advanced or Metastatic Urothelium Cancer	DRUG: MRG002|DRUG: Docetaxel injection|DRUG: Paclitaxel injection|DRUG: Gemcitabine hydrochloride for injection|DRUG: Pemetrexed disodium injection	Shanghai Miracogen Inc.
NCT05445778	Mirvetuximab Soravtansine With Bevacizumab Versus Bevacizumab as Maintenance in Platinum-sensitive Ovarian, Fallopian Tube, or Peritoneal Cancer (GLORIOSA)	https://clinicaltrials.gov/study/NCT05445778 (accessed on 2 September 2024)	Recruiting	Ovarian Cancer|Peritoneal Cancer|Fallopian Tube Cancer	DRUG: Mirvetuximab soravtansine plus bevacizumab|DRUG: Bevacizumab	ImmunoGen, Inc.
NCT02785900	Vadastuximab Talirine (SGN-CD33A; 33A) Combined With Azacitidine or Decitabine in Older Patients With Newly Diagnosed Acute Myeloid Leukemia	https://clinicaltrials.gov/study/NCT02785900 (accessed on 2 September 2024)	Terminated	Acute Myeloid Leukemia	DRUG: 33A|DRUG: Placebo|DRUG: Azacitidine|DRUG: Decitabine	Seagen Inc.
NCT06132958	MK-2870 in Post Platinum and Post Immunotherapy Endometrial Cancer (MK-2870-005)	https://clinicaltrials.gov/study/NCT06132958 (accessed on 2 September 2024)	Recruiting	Endometrial Cancer	BIOLOGICAL: MK-2870|DRUG: Doxorubicin|DRUG: Paclitaxel	Merck Sharp & Dohme LLC
NCT02573324	A Study of ABT-414 in Participants With Newly Diagnosed Glioblastoma (GBM) With Epidermal Growth Factor Receptor (EGFR) Amplification	https://clinicaltrials.gov/study/NCT02573324 (accessed on 2 September 2024)	Completed	Glioblastoma|Gliosarcoma	DRUG: Temozolomide|DRUG: Depatuxizumab mafodotin|RADIATION: Radiation|DRUG: Placebo for ABT-414	AbbVie
NCT03262935	SYD985 vs. Physician’s Choice in Participants With HER2-positive Locally Advanced or Metastatic Breast Cancer	https://clinicaltrials.gov/study/NCT03262935 (accessed on 2 September 2024)	Completed	Metastatic Breast Cancer	DRUG: (Vic-)Trastuzumab duocarmazine|DRUG: Physician’s choice	Byondis B.V.
NCT04924699	A Study of MRG002 in the Treatment of Patients With HER2-positive Unresectable Locally Advanced or Metastatic Breast Cancer	https://clinicaltrials.gov/study/NCT04924699 (accessed on 2 September 2024)	Recruiting	Advanced Breast Cancer|Metastatic Breast Cancer	DRUG: MRG002|DRUG: Trastuzumab emtansine for injection	Shanghai Miracogen Inc.
NCT05950945	Trastuzumab Deruxtecan (T-DXd) in Patients Who Have Hormone Receptor-negative and Hormone Receptor-positive HER2-low or HER2 IHC 0 Metastatic Breast Cancer	https://clinicaltrials.gov/study/NCT05950945 (accessed on 2 September 2024)	Recruiting	Breast Cancer	DRUG: Trastuzumab deruxtecan	Daiichi Sankyo
NCT05329545	Upifitamab Rilsodotin Maintenance in Platinum-Sensitive Recurrent Ovarian Cancer (UP-NEXT)	https://clinicaltrials.gov/study/NCT05329545 (accessed on 2 September 2024)	Terminated	High Grade Serous Ovarian Cancer|Fallopian Tube Cancer|Primary Peritoneal Cancer	DRUG: Upifitimab rilsodotin|OTHER: Placebo	Mersana Therapeutics
